# Perception of COVID-19 Vaccination and Uptake Willingness in Northern Nigeria: Understanding Strategies to Optimize Coverage

**DOI:** 10.3390/ijerph22020153

**Published:** 2025-01-23

**Authors:** Chris Chukwunyere Njoku, Judith Ifunanya Ani, Kezia Batisai

**Affiliations:** 1 Inspire World International Foundation, Abuja 961102, Nigeria; 2 Faculty of Health Sciences, Walter Sisulu University, Mthatha 5117, South Africa; 3 Department of Sociology, Faculty of Humanities, University of Johannesburg, Johannesburg 2006, South Africa

**Keywords:** COVID-19, vaccine uptake, vaccine hesitancy, vaccination perception, northern Nigeria

## Abstract

**Background:** Vaccination is a proven and safe method for combating COVID-19; however, coverage remains low in many low- and middle-income countries, including Nigeria. There is also a lack of contextual evidence regarding the public perception of and willingness to receive vaccines. This study aims to contribute to efforts to optimize the vaccination coverage and improve public health in northern Nigeria. **Objective:** This study investigated the perceptions of COVID-19 vaccination and willingness to uptake the vaccine in northern Nigeria, aiming to identify strategies for optimizing coverage and enhancing vaccination rates. **Methods:** A cross-sectional household survey was conducted across the Federal Capital Territory and five northern states in Nigeria. The survey assessed the respondents’ perceptions of COVID-19, their knowledge of the COVID-19 vaccine, and their willingness to be vaccinated. Descriptive and inferential (multivariate logistic regression) statistical analyses were employed to characterize the population and identify predictors of vaccine uptake. **Results:** The mean age of the respondents was 28.00 years. The majority reported at least one piece of misinformation regarding COVID-19. While awareness of the vaccine was high, its actual uptake was low, and future willingness to receive the vaccine was also limited. Employment status emerged as a significant predictor of vaccine uptake, as determined through the multivariate analysis. Furthermore, collaboration with NGOs and community-based organizations (CBOs) was identified as the most effective strategy for enhancing vaccine uptake. **Conclusions:** This study found a concerningly low willingness to receive the COVID-19 vaccine among participants in northern Nigeria. To address this issue, we recommend establishing partnerships with NGOs and CBOs, implementing extensive public awareness campaigns, and conducting community outreach programs. These efforts should focus on dispelling misinformation, building community trust, and promoting vaccine uptake through culturally appropriate communication materials.

## 1. Introduction

The COVID-19 pandemic, caused by the coronavirus, had a devastating impact on public health, leading to significant challenges for many industrialized and developing nations [1,2]. As of April 2021, barely a few months after the virus broke out, over 4.2 million cases had been confirmed, and about 113,000 deaths were recorded globally. By 1 January 2023, the virus had caused over 12 million confirmed cases and 256,524 deaths, indicating the continued seriousness of the virus, with high morbidity and mortality effects. Of particular concern is Africa’s vulnerability to the health and economic risks posed by the pandemic [3].

To address the pandemic, several biotech and pharmaceutical companies developed COVID-19 vaccines, some of which were approved by the World Health Organization (WHO), including Pfizer-BioNTech, AstraZeneca, Moderna, Sinopharm, Sinovac, and Johnson & Johnson [4]. Nigeria, for example, received 4 million doses of the AstraZeneca/Oxford vaccine in March 2021 through the COVID-19 Vaccines Global Access (COVAX) facility, a collaboration between the Coalition for Epidemic Preparedness Innovations (CEPI), the Global Alliance for Vaccines and Immunizations (GAVI), the United Nations Children’s Emergency Fund (UNICEF), and the WHO [5,6]. This vaccination program prioritized frontline healthcare workers, strategic leaders, security officials, and other eligible public personnel for the first round of vaccinations in March 2021 through the state’s Primary Health Care Board [7].

The World Health Organization African Region posted on Viral Facts Africa’s Facebook page on 10 March 2023 that while the world was slowly returning to normal and COVID-19 restrictions had been lifted, the virus still posed a threat, as people continued to become sick, be hospitalized, and die from COVID-19. As of March 2023 alone, over 135,000 deaths had been recorded globally. This report also highlighted that many people remained unvaccinated, with only 29% of Africa’s population fully vaccinated. The Africa Centre for Disease Control, in its 5 February 2023 update, reported that only 49.7% full vaccination coverage had been achieved, indicating the low vaccination coverage across the continent. In Nigeria, only 59.3% of the eligible population had been fully vaccinated out of 115,983,921 eligible individuals^3^. This low vaccine uptake implies that the virus may develop new variants and evade the immunity offered by vaccines, thereby prolonging the pandemic.

Vaccine hesitancy is dangerous not only to individual health but also to society at large, slowing uptake and hindering the achievement of herd immunity [8]. Scholars have identified various reasons for vaccine hesitancy, including confidence, convenience, and complacency, referred to as the “3Cs” [9,10]. Confidence relates to trust in the safety, effectiveness, and competence of health professionals and the healthcare system, as well as trust in policymakers’ motives for the vaccine. Convenience refers to the availability, accessibility, and affordability of and willingness to pay for the vaccine. Complacency relates to the perceived low risk of contracting the virus, which reduces the perceived need for vaccination. Other determinants of vaccine hesitancy include contextual factors, individual and group influences, and vaccine/vaccination-specific issues [11].

Vaccine hesitancy is a significant public health challenge that refers to a delay to taking or refusal to take vaccines, even when they are available [10,12]. In Nigeria, the COVID-19 vaccination program has encountered several challenges, including vaccine hesitancy resulting from misinformation. The historical antecedents of vaccine hesitancy in Nigeria can be traced back to the misinformation and boycott associated with the polio vaccination program in 2003 in northern Nigeria, which had devastating outcomes for the population [13,14]. During the polio vaccine rollout, the public feared that the vaccine was unsafe due to rumors spread by certain northern Nigerian leaders who claimed the vaccine contained HIV, cancerous agents, and anti-fertility agents aimed at reducing the population size [15]. This lack of public trust persisted for a long time and significantly affected the polio vaccination program.

Similar suspicions and distrust have now surfaced regarding the COVID-19 vaccine. People believe in various forms of misinformation, including claims that the virus is a biological weapon from China or the West, aimed at reducing the world’s population. Conspiracy theories and misinformation have been known to halt global efforts to eradicate diseases, as was the case with the polio vaccination campaign in northern Nigeria.

In his 2005 article “A Conspiracy Theory Spreads Polio”, Pipes [15] documented how conspiracy theories hindered global efforts to eradicate polio, leading to an increase in the number of cases worldwide. He cited the views of an Islamist, Dr. Ibrahim Datti Ahmed, who likened the polio vaccination campaign to the war in Iraq, arguing that America’s fight against the Middle East was akin to a war against Muslims. Additionally, Ibrahim Shekarau, the then-governor of Kano state in northern Nigeria, infamously declared that sacrificing a few children was preferable to allowing millions of girl-children to become infertile, as he believed the vaccine contained harmful agents. These attitudes fueled vaccine hesitancy in northern Nigeria, with many cultural and religious leaders opposing the vaccination campaigns. It was a daunting task to convince the public of the vaccine’s safety and efficacy [13].

From a sociological perspective, many of these suspicions and misinformation are not isolated phenomena. Other factors that contributed to suspicion included the 1980 Population Policy, which advocated for a limit of four children per woman, and the controversial 1996 Pfizer trovafloxacin (Trovan) trial. These events increased public distrust in health interventions and amplified the resistance to polio vaccines. As a result, by 2012, Nigeria accounted for more than half of all polio cases worldwide. Although significant success has been achieved in combating polio, vaccine hesitancy remains a significant challenge, reflecting how difficult it is to persuade the population to trust the safety and importance of vaccines [16].

The advent of 5G technology, coinciding with the coronavirus pandemic, further fueled doubts about the virus and the COVID-19 vaccine, contributing to vaccine hesitancy. Additionally, widespread distrust of the government due to failed promises and perceived neglect during the COVID-19 relief efforts may have played a role. Many people hypothesized that a government that watched its citizens suffer from a “hunger pandemic” could not genuinely care about their health. This mistrust likely contributed to the challenges surrounding vaccine uptake in Nigeria.

While studies have been conducted to understand vaccine hesitancy in Nigeria [17,18,19,20], there is limited knowledge about vaccine hesitancy in northern Nigeria, a region that has experienced vaccine resistance in the past. Addressing the concerns of the population and communicating the importance of vaccine safety are essential to ensuring that the population is adequately vaccinated against COVID-19. As Nigeria is a multi-ethnic and multi-religious country with diverse cultural contexts, understanding the specific reasons behind public attitudes toward vaccines is key to effective interventions. Thus, this study aims to determine strategies to optimize coverage by understanding the perception and uptake willingness for COVID-19 vaccination in northern Nigeria.

## 2. The Theoretical Framework

This study employs the Health Belief Model (HBM) as its theoretical framework to understand the factors influencing the perception of COVID-19 vaccination and uptake willingness in northern Nigeria. The HBM, developed by social psychologists in the 1950s to explain health-related behaviors, posits that individual decisions about health actions are influenced by personal beliefs about disease threat, the benefits of taking action, and barriers to action. This model is particularly relevant to vaccination behaviors, where decisions are shaped by perceptions of susceptibility, severity, benefits, barriers, cues to action, and self-efficacy. This theoretical application highlights key constructs that influence the perception of COVID-19 vaccination and uptake willingness. Perceived susceptibility refers to individuals’ beliefs about their likelihood of contracting COVID-19, with low perceived risk as a significant contributor to vaccine hesitancy. Similarly, perceived severity captures beliefs about the seriousness of the disease, with misconceptions—such as the belief that COVID-19 is a scam or less harmful in Nigeria’s hot climate—diminishing the perceived urgency of vaccination. Perceived benefits refer to trust in the vaccine’s ability to control the virus. Conversely, perceived barriers include fears about the side effects of the vaccine and distrust in government initiatives. Additionally, cues to action, such as trusted information from NGOs, community-based organizations (CBOs), and traditional or religious leaders, constitute critical triggers for vaccination. Lastly, self-efficacy, which in this study refers to individuals’ confidence in their ability to take preventive action, is vital to overcoming hesitancy. This model provides a comprehensive framework for understanding the psychosocial and contextual factors that influence COVID-19 vaccination behavior in northern Nigeria. By integrating its key constructs, this study highlights how perceptions of susceptibility, severity, benefits, and barriers shape individual decisions, while cues to action and self-efficacy provide critical pathways to behavior change. This theoretical lens therefore underscores the interplay between individual beliefs and external influences, such as trust in healthcare systems and community organizations, which are pivotal in addressing vaccine hesitancy. The application of the HBM not only clarifies the determinants of vaccine hesitancy but also informs targeted interventions. Strategies derived from this framework, such as leveraging trusted community voices, dispelling misinformation, and fostering confidence in vaccine efficacy and safety, align with its constructs and can significantly enhance vaccination uptake. Thus, the HBM serves as both a diagnostic tool for identifying barriers and a prescriptive guide for designing culturally sensitive and evidence-based interventions aimed at achieving the optimal vaccination coverage.

## 3. Materials and Methods

Study design: This was a cross-sectional survey. It was an interviewer-administered, closed-ended questionnaire that was incorporated into the Kobo Collect survey tool.

Study setting: This study was conducted across the Federal Capital Territory and five (5) states in northern Nigeria (Abuja, Adamawa, Bauchi, Nasarawa, Plateau, and Taraba states). To ensure spread and support from the respondents, research assistants who were also young community influencers were recruited, trained, and equipped with resources and engaged to collect data across various communities in these states.

Study population and inclusion criteria: This study included individuals aged 18 years and above who were residents of these states irrespective of gender, socioeconomic status, educational background, or religious affiliation.

Sampling and sample size calculation: Respondents were recruited using a simple random sampling method. This ensured that all eligible individuals within the targeted states had an equal chance of being selected. To establish a representative sampling frame, the communities within each state were mapped, and households were randomly selected using a structured approach. The research assistants, who were trained and familiar with the local context, visited the selected households to recruit eligible participants aged 18 years and above. This process ensured that individuals from various demographic and socioeconomic backgrounds were included. By adhering to random selection principles within the identified communities, the sampling process minimized the selection bias and enhanced representativeness. For a population of 25 million Nigerians across the sampled northern states, a minimum sample size of 664 was computed at a 99% confidence level, a 5% margin of error, and a 50% response rate based on the Open-Source Epidemiologic Statistics for Public Health (OpenEpi), version 3 [21,22]. In addition to the sample size and to account for potential non-responses, an attrition rate of 10% was added, resulting in a target sample size of 730. Ultimately, 715 datasets were retrieved and analyzed. This multistaged approach ensured that the study findings reflected the population surveyed and provided robust insights into vaccine perceptions and the willingness to be vaccinated in northern Nigeria.

Data collection and analysis: A closed-ended questionnaire was used to elicit responses from the respondents. It was designed in the English language, and it took an average of 10 min to complete. Content validity was established through an extensive review of the relevant literature. Input from public health and sociology experts was also sought to ensure the items comprehensively captured the dimensions of COVID-19 vaccine awareness, perception, and willingness. Additionally, face validity was achieved by pre-testing the questionnaire among a small, representative sample of the target population. This step allowed us to assess the clarity, cultural appropriateness, and relevance of the questions. Feedback from the pre-testing process informed refinements to the wording and structure of the items, ensuring they aligned with this study’s objectives and were easily understood by the respondents. Test–retest reliability was also adopted in this study to ensure that the responses were similar across different time intervals. The participants were assured of its confidentiality. The concepts of voluntary participation, benefits, non-malfeasance, and withdrawal were explicitly explained to the respondents. The e-survey tool contained sociodemographic questions on factors such as age, gender, marital status, income, employment, and economic status. Household economic status was derived based on a household asset ownership classification. Household economic status was grouped into three levels: low, average, and high. Those who owned 3 assets or below were classified as low; those who owned between 3 and 6 assets were classified as average; and households with more than 6 were classified as high. Responses were primarily measured using a combination of categorical variables (e.g., “Yes,” “No”) and Likert scales (e.g., “Strongly Agree” to “Strongly Disagree”). Information on misinformation was elicited and construed on a five-point Likert scale (Strongly Agree, Agree, Neutral, Strongly Disagree, and Disagree). Vaccine knowledge was elicited among the respondents, and the variables for this measure varied. This study also elicited information on willingness in terms of vaccine uptake by asking whether the respondents would take the vaccine or not and their reasons. The respondents were also asked about their likelihood of taking the vaccine in the future.

Data were extracted from the e-survey tool, sorted, and analyzed using the IBM SPSS version 24.0 software. A total of 715 questionnaires were analyzed after eliminating incomplete questionnaires. Descriptive statistics (frequencies and percentages) were used to analyze the sociodemographics and categorical variables. Continuous variables were analyzed using the means and standard deviations. Logistic regression was used to determine the odds ratios for the associations between the sociodemographic variables and uptake willingness. *p*-values of less than 0.05 and 95% confidence intervals were considered significant. The variables included in the regression model were selected based on their theoretical relevance to vaccine uptake willingness, as informed by the existing literature and this study’s objectives. Sociodemographic variables, such as age, gender, employment status, and area of residence, were included to explore their influence on vaccine uptake willingness. These variables are commonly associated with health behaviors and were deemed essential for understanding the factors influencing vaccination decisions. The adjusted odds ratios (AORs) derived from the multivariate logistic regression model accounted for the influence of all of the variables included, providing insights into the independent effect of each predictor on vaccine uptake willingness while controlling for potential confounding factors.

### Ethical Considerations

This study was approved by the National Health Research and Ethics Committee of Nigeria (NHREC) (NHREC/01/01/2007-11/05/2023B). All of the respondents provided written informed consent to take part in this study. The methods were carried out in accordance with the relevant guidelines and regulations. The data gathered for this study were stored in a secured database that was accessible to the authors only.

## 4. Results

The sociodemographic characteristics of the respondents, as presented in Table 1, reveal a comprehensive overview of the study population. A total of 715 forms were analyzed, and a larger proportion of the respondents were male (58.6%) compared to female (41.4%). The mean age of the respondents was 28 years, with a significant portion (44.6%) of them falling within the 18–25 age group. Most of the respondents had completed secondary education (35.0%), while 32.0% had attained tertiary education. The majority were single (78.6%), and 19.6% were married. Among those married, monogamous unions were predominant (80.4%).

Regarding their economic status, 60.7% of the respondents reported having an average household economic standing, while 16.1% indicated a low status. Only 19.3% of the respondents were employed, while 38.5% were self-employed, and a notable proportion (42.2%) were unemployed. In terms of the income distribution, over half of the respondents (54.4%) had a monthly income ranging between NGN 20,001 and NGN 40,000, and 35.5% earned NGN 20,000 or less.

Concerning health-related factors, only 7.7% of the respondents had been diagnosed with COVID-19, while the vast majority (91.3%) reported having no underlying health conditions. Additionally, the data show that the majority of the respondents lived in rural areas (81.0%), with smaller percentages residing in semi-urban (9.9%) and urban areas (9.1%).

### 4.1. COVID-19 Misinformation

This study examined the extent of COVID-19 misinformation among the respondents, and the findings revealed some concerning trends. As presented in Table 2 below, a significant proportion held false beliefs about the virus, with one of the most widespread misconceptions being that COVID-19 was a biological weapon intentionally released by China. A notable 45.1% of the respondents agreed with this idea, reflecting the influence of conspiracy theories during the pandemic. Similarly, 38.5% believed that the virus was used as a tool to reduce the global population. These narratives, often rooted in geopolitical tensions, highlight how misinformation can skew public understanding of major global health events.

Further deepening these concerns was the finding that 34.2% of the respondents believed the virus was a scam within Nigeria, suggesting a strong undercurrent of skepticism toward the reality of COVID-19 in the country. Additionally, 34.0% thought the virus was being used by the government to steal money. This reflects a broader distrust of public institutions, which could have far-reaching consequences for public health compliance and government-led interventions, such as vaccination campaigns or lockdowns. These beliefs are likely to have affected how seriously the respondents took COVID-19 safety measures, ultimately impeding efforts to curb the virus’s spread.

This study also uncovered several misconceptions related to traditional health practices and environmental conditions. For instance, 20.9% of the respondents believed that chewing raw ginger and garlic could prevent or treat the virus, while 37.7% thought the virus could not survive in areas with hot climates. These beliefs indicate how local health traditions and misunderstandings about the virus’s behavior in different climates may have steered people away from scientifically proven methods for prevention and treatment. Such misinformation may have contributed to the lower adherence to appropriate medical guidelines, increasing the population’s vulnerability to the virus.

The misconception linking 5G technology to the spread of COVID-19, though it was less widespread, still appeared among 19.6% of the respondents. This finding demonstrates how technological advancements can become intertwined with health misinformation, fostering fear and mistrust of new technologies. Beliefs like these can lead to destructive behavior, as seen in some parts of the world, where 5G infrastructure was vandalized during the pandemic.

Overall, this study found that nearly 29% of the respondents held at least one belief in misinformation about COVID-19, indicating a pervasive spread of false information. The combination of geopolitical conspiracy theories, distrust in the government, reliance on traditional remedies, and misunderstanding of technological and environmental factors underscores the urgent need for targeted public health communication. Addressing these misconceptions is critical to building public trust and ensuring that future health interventions are effective and widely adopted.

### 4.2. COVID-19 Vaccine Knowledge

The respondents’ knowledge of the COVID-19 vaccine revealed important insights into the public understanding of vaccination, as show in Table 3 below. While 76.2% of the respondents were aware of the COVID-19 vaccine, their sources of information varied. Television (19.1%) and community outreach programs (18.6%) were the primary channels through which they learned about the vaccine, followed closely by social media (17.3%), reflecting the significant role of mass and digital media in spreading vaccine-related information. However, despite widespread awareness, only 37.8% of the respondents believed that the vaccine could effectively control the virus. This indicates a gap between awareness and trust in the vaccine’s efficacy, which could hinder vaccination efforts.

Another critical finding was that the large majority (59.7%) of the respondents did not know how many doses were required for proper vaccination, which points to a lack of detailed knowledge about the vaccination process. This lack of clarity may contribute to confusion and hesitation about receiving the vaccine, particularly when respondents are uncertain about what constitutes full immunization. Additionally, there was significant variation in the respondents’ opinions on which age and occupational groups should be prioritized for vaccination, indicating an inconsistent understanding of the vaccine distribution strategies.

Concerns about the vaccine’s side effects were prevalent, with 62.5% of the respondents believing that the vaccine could have adverse effects. Of those, 21% believed the side effects would be mild, such as headaches, fever, or nausea, while 10.2% thought the side effects could be serious and life-threatening. Such concerns might increase vaccine hesitancy and highlight the need for clear communication regarding the safety and side effect profile of COVID-19 vaccines.

When asked which age group should be prioritized for vaccination, 51% of the respondents believed that everyone, regardless of age, should be vaccinated. This perspective aligns with the broader goal of achieving widespread immunity. However, a portion of the respondents prioritized specific age groups, with younger adults (11.2%) and older adults (11.2%) receiving the most support. This variability suggests that while there is general support for vaccination, targeted education on the most vulnerable populations could help refine public understanding.

In terms of occupational prioritization, 27.3% of the respondents correctly identified healthcare workers as the group most in need of early vaccination. However, there was notable uncertainty, with 26.2% of the respondents unsure of which occupational groups should be prioritized. Other groups, such as hospitalized patients (13.6%) and travelers (9.1%), were also mentioned but to a lesser extent. This highlights the need for clearer messaging regarding vaccination strategies to ensure the public understands which populations are most at risk and should be prioritized for vaccination.

Overall, these findings reveal that while awareness of the COVID-19 vaccine is high, there is still a considerable gap in detailed knowledge about its efficacy, the dosing regimen, and vaccination prioritization. Addressing these gaps through targeted communication efforts could improve vaccine acceptance and ensure a more informed public.

### 4.3. COVID-19 Vaccine Uptake Willingness/Hesitancy and Implementation Strategies

The respondents were asked about their willingness to take the COVID-19 vaccine, revealing significant levels of hesitancy. As shown in Table 4 below, only 32.7% affirmed their willingness to be vaccinated, with the majority (67.3%) stating they would not take the vaccine. The main reasons for their hesitancy included the belief that they were not at risk of contracting the virus (31.9%) and concerns about the safety of the vaccine (26.2%). These findings highlight widespread misconceptions about individual vulnerability to COVID-19 and persistent doubts about vaccine safety.

Further probing into their future vaccine uptake revealed even lower optimism. A striking 93.4% of the respondents expressed no likelihood of taking the vaccine in the future, while only 6.6% affirmed they would likely take it. This overwhelming reluctance to consider future vaccination underscores a deep-seated resistance that could pose significant challenges to public health efforts aimed at achieving herd immunity.

The respondents cited several major challenges to vaccine uptake. Fear of side effects was the most commonly reported barrier (25.5%), followed closely by widespread disbelief about the existence or seriousness of the virus (24.6%) and a general lack of trust in the government (20.0%). These concerns reflect broader societal issues of misinformation, skepticism about the government’s handling of the pandemic, and a lack of trust in health systems and official information. Religious beliefs (9.0%) and logistical issues, such as the cost of the vaccine (7.0%) and problems related to vaccine storage and transportation (7.6%), were also mentioned but to a lesser extent.

When asked how the vaccination program should be implemented, respondents favored non-governmental organizations (NGOs) and community-based organizations (CBOs) as the primary channels for vaccine distribution (44.1%). This preference reflects a possible lack of trust in government-led efforts. Government hospitals were the second most preferred option (39.7%), while private clinics (6.7%) and religious bodies (9.5%) were less favored. These findings suggest that leveraging NGOs and community organizations may increase vaccine uptake by addressing concerns related to trust and accessibility.

These insights reveal the urgent need for targeted education campaigns to dispel myths, address concerns, and build public confidence in both the vaccine and the broader COVID-19 response. The reliance on trusted local organizations, such as NGOs and CBOs, could be key to overcoming hesitancy and improving the vaccine distribution, particularly in communities with low trust in government efforts.

### 4.4. A Multivariate Analysis of Sociodemographic Factors Influencing Willingness to Take the COVID-19 Vaccine

A multivariate logistic regression analysis was conducted to determine the influence of the sociodemographic factors on willingness to take the COVID-19 vaccine. The variables included gender, area of residence, marital status, the presence of underlying ailments, a previous diagnosis of COVID-19, employment status, and age. Among these factors, only employment status had a significant association with vaccine uptake willingness (Table 5).

Employment status stood out as a key determinant, with employed individuals having significantly higher odds (AOR = 2.505, *p* < 0.001) of being willing to take the vaccine compared to their unemployed counterparts. This could be attributed to organizational policies that require vaccination for workplace entry, making employment a practical factor driving vaccine uptake. Similarly, self-employed individuals were more likely to take the vaccine (AOR = 1.656, *p* = 0.012), though this association was weaker compared to that for those employed in organizations. Other sociodemographic factors such as gender, area of residence, and a previous diagnosis of COVID-19 did not show significant associations with vaccine uptake. For instance, females were less likely than males to express vaccine uptake willingness (AOR = 0.848), but this association was not statistically significant (*p* = 0.334). Similarly, rural and semi-urban residents showed no significant differences from their urban counterparts, though semi-urban dwellers had higher odds of willingness (AOR = 2.000, *p* = 0.065) that neared significance.

Marital status was also not a significant predictor, though the married respondents had higher odds (AOR = 1.533, *p* = 0.055) of expressing willingness compared to single individuals, with this result approaching statistical significance. Additionally, divorced, separated, or widowed respondents had higher odds of willingness (AOR = 2.455), though this finding was not statistically significant (*p* = 0.135). The presence of underlying ailments significantly influenced vaccine uptake. Respondents without underlying ailments were less likely to be willing to take the vaccine compared to those with health conditions (AOR = 0.513, *p* = 0.022), suggesting that individuals with underlying conditions may perceive themselves as more vulnerable to the virus and thus more inclined to be vaccinated.

Age was a significant predictor of vaccine acceptance for certain groups. Individuals aged 26–35 years had lower odds (AOR = 0.673, *p* = 0.024) of being willing to take the vaccine compared to those aged 18–25. Similarly, respondents aged 36–45 years also had lower odds (AOR = 0.494, *p* = 0.022) of vaccine willingness relative to those of the youngest age group. Both findings were statistically significant. In contrast, individuals aged 46–55 years had lower odds (AOR = 0.692) of vaccine willingness compared to those for the 18–25 age group, though this result was not statistically significant (*p* = 0.330). Additionally, respondents aged 56 and above had slightly higher odds (AOR = 1.169) of vaccine willingness compared to the youngest group, but this finding was also not statistically significant (*p* = 0.794). Overall, this highlights the significantly lower vaccine acceptance among individuals aged 26–45 compared to younger adults (18–25 years). For older age groups (46–55 and 56+), no significant differences in willingness were observed, suggesting similar vaccine acceptance rates to those in the youngest group. These trends may reflect differences in perceived vulnerability, risk assessment, or trust in vaccines across age groups.

The significant influence of employment status on vaccine willingness suggests that institutional policies play a crucial role in encouraging vaccine uptake. This points to the potential effectiveness of workplace-based interventions or policies that incentivize vaccination. Moreover, the finding that individuals with underlying ailments were more willing to take the vaccine highlights the importance of targeted messaging to vulnerable populations who may perceive a greater personal risk of COVID-19. On the other hand, the lack of significant associations with the other sociodemographic variables such as gender, marital status, and area of residence suggests that efforts to increase vaccine uptake need to focus less on these factors and more on addressing misinformation and perceived risk. Specifically, interventions that address safety concerns and provide clear, consistent messaging about the risks of not being vaccinated may be more effective.

## 5. Discussion

The primary aim of this study was to explore the perception of COVID-19 vaccination, uptake willingness, and strategies to optimize the vaccine coverage in Northern Nigeria. Our findings revealed that two years after the virus’s discovery, unsubstantiated claims and misinformation about COVID-19 remained prevalent. This underscores the widespread persistence of erroneous narratives that continue to influence public perception. Our results mirrored the global trends reported by [23], where misinformation and conspiracy theories about the virus were spread across regions, undermining people’s confidence in both COVID-19 response and vaccination efforts. These insights highlight the critical need for evidence-based communication strategies to counter these false narratives and promote public trust in the vaccine and the broader pandemic response.

Misinformation, with its potential for negative health consequences, fosters conspiratorial thinking, which cannot be overlooked in a public health crisis. As the World Health Organization (WHO) [24] pointed out, misinformation acts as an “infodemic”, overwhelming the public with excessive and often incorrect information during disease outbreaks, leading to tragic outcomes. The WHO reported that thousands of people worldwide were hospitalized due to COVID-19 misinformation. In parallel, conspiratorial thinking arises when people believe that powerful actors engage in secretive activities for personal gain [25]. While misinformation often stems from ignorance, conspiracy theories are typically fueled by distrust in governments, suspicion of global actors’ motives, and beliefs in secret plots by elites to manipulate and control the population.

Scholars have noted that misinformation and conspiracy theories thrive during health emergencies and societal crises [26]. In such situations, people are more inclined to accept hoaxes over scientific facts. A study in the U.S. revealed that over 85% of the respondents believed in at least one conspiracy theory related to COVID-19 [27,28]. Common narratives included the belief that COVID-19 was a biological weapon designed to reduce the global population, a theory that was also widely reported in other studies [29,30,31].

Despite the prevalence of misinformation, our study found a high level of awareness about the COVID-19 vaccine in Northern Nigeria. This widespread awareness can be attributed to the efforts of the Nigerian government, in collaboration with organizations such as UNICEF and the National Primary Health Care Development Agency (NPHCDA), as well as other stakeholders. These entities undertook extensive sensitization campaigns, mobilizations, and training programs across the country, particularly regarding vaccine uptake. Their use of multiple communication channels—television, radio, social media, and community outreach—equipped the public with situational awareness and knowledge, which likely contributed to increased vaccine uptake [32,33]. Our findings regarding the sources of COVID-19 vaccination information aligned with those of other studies [34,35,36], which also identified traditional and social media as the dominant sources of information. In Nigeria, social media campaigns on platforms like Facebook and Instagram, featuring frames and stickers such as “I Got My COVID-19 Vaccine” and “Let Us Get Vaccinated”, played a key role in creating awareness and encouraging behavior change [37].

Although this study recorded a high level of awareness about the vaccine, it revealed a gap in the understanding of the correct dosage required for full vaccination. Moreover, there were negative perceptions about the vaccine’s efficacy, with concerns about its ability to prevent the virus. These perceptions were shaped by issues such as distrust in the government, concerns over safety and efficacy, and the belief that individuals were not at risk of contracting the virus. Similar findings were reported in other studies [38,39], which highlighted distrust in the government, uncertainty about the vaccine, fear of side effects, and doubts about the vaccine’s efficacy and safety as key reasons for vaccine hesitancy.

When asked about their future likelihood of taking the COVID-19 vaccine, the majority of the respondents expressed uncertainty, citing disbelief in the virus, distrust in the government, and fear of side effects. These responses underscore the persistence of vaccine hesitancy in the region. This study’s analysis revealed that employment status was the most significant predictive factor for vaccine uptake willingness. Respondents who were employed or self-employed were more willing to be vaccinated compared to the unemployed. This association might be due to workplace policies requiring vaccination for access to certain spaces or job security. However, the overall uncertainty and hesitancy among the respondents highlight the need for more targeted vaccine campaigns that emphasize the safety and efficacy of the vaccine. Scholars have warned that vaccine hesitancy poses a serious threat to society by lowering herd immunity, particularly among vulnerable populations such as the elderly and those with weakened immune systems [40].

## 6. Conclusions and Recommendations

This study aimed to enhance our understanding of the perception of COVID-19 vaccination, uptake willingness, and strategies for optimizing coverage in Northern Nigeria. Its findings revealed a high level of awareness about the vaccine but a low willingness to be vaccinated and considerable uncertainty regarding future uptake. The data identified several barriers to vaccination, including government distrust, concerns over safety and efficacy, fear of side effects, and pervasive misinformation.

To optimize vaccination coverage and uptake willingness, it is essential for Nigeria’s health system to reevaluate its response to public health emergencies that necessitate vaccination interventions. It is essential to address misinformation and build culturally appropriate messaging that fosters community trust and acceptance. Addressing issues of trust is critical to fostering improved acceptance of interventions supported by government healthcare providers. Strengthened collaboration with non-governmental organizations (NGOs) and community-based organizations (CBOs) is also viewed as a potential solution for enhancing local acceptance of health interventions. Policymakers and the government should ensure the continuous dissemination of targeted COVID-19 messages through channels identified as acceptable by the community. Achieving herd immunity is paramount, necessitating extensive vaccination campaigns. Such efforts should include ongoing consultation with traditional and religious leaders, integrating them at every implementation level to foster community support. In this region, it is crucial to ensure the full participation of religious, traditional, and political leaders. COVID-19 vaccination campaigns should employ culturally relevant media communications that resonate with the community, utilizing local languages to engage both the intellect and emotions of the populace.

## 7. Limitations

This study has several limitations. As a cross-sectional study, it provides only a snapshot of data at a specific point in time, limiting the ability to observe changes in attitudes and behaviors over time. A longitudinal approach would be more suitable for capturing such dynamics. Additionally, although this study included six states in Northern Nigeria, its findings may not be fully generalizable to the entire Nigerian population due to regional variations in cultural, socioeconomic, and demographic factors. There was also the potential for self-selection bias, as individuals who voluntarily participated may have had different views on vaccination compared to those who opted not to participate.

Despite these limitations, this study makes important contributions by shedding light on the critical barriers to COVID-19 vaccination uptake in Northern Nigeria. It highlights key factors such as government distrust, misinformation, and concerns about vaccine safety and efficacy, offering valuable insights for targeted interventions. By identifying these challenges, this study provides a foundation for developing more effective, culturally relevant communication strategies and collaborations with community-based organizations to improve the vaccine coverage in this region.

## Figures and Tables

**Table 1:** Sociodemographic characteristics of respondents.

Variables		Frequency N = 715	Percentage (%)
Gender	Male	419	58.6
	Female	296	41.4
Area	Rural Semi-urban Urban	579 71 65	81.0 9.9 9.1
Age Mean age = 28.00 years Standard deviation: 9.247	18 to 25 years	319	44.6
	26 to 35 years	278	38.9
	36 to 45 years	69	9.7
	46 to 55 years	37	5.2
	56 years and above	12	1.7
Highest level of education	No formal education	15	2.1
	Primary	221	30.9
	Secondary	250	35.0
	Tertiary	229	32.0
Type of marital union (for married respondents only)	Monogamy	575	80.4
	Polygamy	93	13.0
Household economic status	Low	115	16.1
	Average	434	60.7
	High	80	11.2
	No response	86	12.0
Employment status	Employed	138	19.3
	Self-employed	275	38.5
	Unemployed	302	42.2
Average monthly income (in naira)	0–20,000	254	35.5
	20,001–40,000	389	54.4
	40,001–60,000	24	3.4
	60,001–80,000	18	2.5
	80,001–100,000	13	1.8
	100,001–120,000	4	0.6
	120,001–140,000	1	0.1
	140,001 and above	12	1.7
Any underlying ailments?	Yes	62	8.7
	No	653	91.3
Prior diagnosis of COVID-19?	Yes	55	7.7
	No	610	85.3
	No response	50	7.0

**Table 2:** Percentage distribution of respondents by misinformation regarding COVID-19.

Variables	Agree	Neutral	Disagree
The virus is a biological weapon intentionally released by China	45.1	26.3	28.6
The virus is a biological weapon intentionally released by the United States of America	18	29.4	52.6
The virus is not real in Nigeria	34.2	22	43.7
The virus is a scam used by the government to steal money	34.0	26.4	39.6
The virus is a biological weapon used to reduce the population of the world	38.5	30.8	30.7
Anybody can prevent or treat the virus by chewing raw ginger and garlic	20.9	31.3	47.8
The virus cannot survive in areas with a hot climate	37.7	32.9	29.4
5G technology is used to spread COVID-19	19.6	39.7	40.7
COVID-19 no longer exists	29.7	27	43.4
Antibiotics and antimalarial are used to treat and prevent the virus	19.1	35.2	45.7
People with the virus recover without any treatment	20.0	31.6	48.4
**Average responses**	28.8	30.2	41.0

**Table 3:** Percentage distribution of respondents by COVID-19 vaccine knowledge.

Variables	Percentage (%)
**Heard/know about the COVID-19 vaccine**	
Yes	76.2
No	23.8
**Sources of knowledge of the COVID-19 vaccine**	
Television	19.1
Social media	17.3
Radio	10.8
Friends/colleagues	12.4
Family	11.3
Community outreach	18.6
Healthcare centers/hospitals	10.6
**Believe that the vaccines can prevent the virus**	
Yes	37.8
No	62.2
**How many doses required for proper vaccination**	
Do not know	59.7
One dose	6.9
Three doses	19.3
Two doses	14.1
**COVID-19 vaccine has side effect**	
Yes	62.5
No	37.5
**Types of side effects the vaccine has**	
Not applicable *	62.5
Do not know	6.3
Mild or not serious side effects (e.g., headache, fever, nausea, etc.)	21.0
Serious side effects (e.g., life-threatening)	10.2
**Age groups to be prioritized for vaccination**	
Children (0–10 years)	5.2
Adolescents (11–17 years)	10.7
Younger adults (18–29 years)	11.2
Adults (30–59 years)	5.2
Older adults (60 years and above)	11.2
All	51.0
Not sure	5.5
**Occupational groups to be prioritized for vaccination**	
Health workers	27.3
Hospitalized patients	13.6
Travelers	9.1
Traders/business men/women	6.4
Civil servants	5.5
Students	5.0
Public servants	3.8
Lecturers/teachers	3.2
Not sure	26.2

* The proportion of respondents who said “No” to the question “COVID-19 vaccine has side effect”.

**Table 4:** Percentage distribution of respondents by COVID-19 vaccine uptake willingness/hesitancy and implementation strategy.

Variables	Frequency (%)
Will you take the COVID-19 vaccine?	
No	67.3
Yes	32.7
**Reasons for hesitancy**	
It is deadly	8.9
It is not effective	12.8
It is not safe	26.2
It is costly	5.7
More studies needed to validate the vaccine	14.5
Not at risk of contracting the virus	31.9
**Likelihood that you will take the COVID-19 vaccine in future**	
No likelihood at all	93.4
Very likely	6.6
**Major challenge for vaccine uptake**	
Cost of the vaccine	7.0
Lack of motivation/incentive to receive the vaccine	6.3
Logistics problems (transportation, vaccine storage)	7.6
Fear of side effects	25.5
People’s disbelief about the virus	24.6
People’s lack of trust in the government	20.0
Religious beliefs and opinions	9.0
**How vaccination programme should be implemented**	
Through government hospitals	39.7
Through NGOs/CBOs	44.1
Through private clinics	6.7
Through religious bodies	9.5

**Table 5:** Sociodemographic factors influencing uptake willingness for the COVID-19 vaccine.

Variables	Categories	Coeffi (B)	S.E	*p*-Value	Adjusted Odds Ratio (Exp (B)	95% Confidence Interval	
						Lower Bound	Upper Bound
Gender	Female	−0.165	0.171	0.334	0.848	0.607	1.185
	Male (R)						
Area	Rural	0.139	0.298	0.641	1.149	0.641	2.060
	Semi-Urban	0.693	0.376	0.065	2.000	0.957	4.177
	Urban (R)						
Marital status	Divorced/Separated /Widowed	0.898	0.601	0.135	2.455	0.756	7.970
	Married	0.427	0.222	0.055	1.533	0.991	2.370
	Single (Never Married) (R)						
Underlying ailments?	No	−0.668	0.291	0.022	0.513	0.290	0.907
	Yes (R)						
Previously diagnosed with COVID-19?	No	−0.280	0.311	0.368	0.756	0.410	1.391
	Yes (R)						
Employment status	**Employed**	0.918	0.240	0.000	2.505	1.567	4.006
	**Self-Employed**	0.505	0.200	0.012	1.656	1.119	2.452
	Unemployed (R)						
Age	18–25 Years (R)						
	26–35 Years	−0.396	0.175	0.024	0.673	0.478	0.948
	36–45 Years	−0.705	0.308	0.022	0.494	0.270	0.904
	46–55 Years	−0.368	0.378	0.330	0.692	0.330	1.452
	56 Years and Above	0.156	0.597	0.794	1.169	0.363	3.766

## Data Availability

The raw data supporting the conclusions of this article will be made available by the authors on request.
